# Supplementary material for: What are the effects of teaching Evidence-Based Health Care (EBHC) at different levels of health professions education? An updated overview of systematic reviews
Source: PLoS One. 2021 Jul 22;16(7):e0254191. doi: 10.1371/journal.pone.0254191 (PMC8297776; doi:10.1371/journal.pone.0254191)
Supplement: S1 File — (DOC) [file pone.0254191.s003.doc]

# Effects of teaching Evidence-based Health Care to under- and postgraduate students in the health professions: Overview of systematic reviews

**Faculty of Medicine and Health Sciences, Stellenbosch University**

**PROPOSAL**

Young T*, Rohwer A, Poklepović Peričić T, Bala MM

*Contact details

Dr T Young

MB,ChB, FCPHM, MMED (Public Health)

Centre for Evidence-based Health Care

Faculty of Medicine and Health Sciences

Stellenbosch University

Email: [tyoung@sun.ac.za](mailto:tyoung@sun.ac.za)

Tel: +27 21 938 9452/9157

Initial overview date: **10 October 2012**

Update: **12 February 2020**

**Contents**

1.Synopsis 3

2. Background 5

3. Objectives 7

4. Methods 7

5. Contribution of authors 11

6. Declarations of interest 11

7. References 11

8. Sources of support 12

9. Appendix 1 13

# SYNOPSIS

***Title***

# Effects of teaching Evidence-based Health Care to under- and postgraduate students in the health professions: Overview of systematic reviews

***Introduction and Literature review***

An evidence-based approach to healthcare is recognized internationally as a key competency for healthcare practitioners. It is recommended that EBM becomes a core part of the curriculum of all healthcare professionals, since learning the fundamentals of research and the basic knowledge and skills of EBM are essential for successful implementation of EBHC and subsequent improvement in quality of healthcare and health outcomes (Glasziou 2008). However, despite the recognition of EBHC as a key competency, the level of EBHC teaching and learning at both student and professional levels is often haphazard, fragmented or non-existent. Where offered, input is conducted as isolated teaching sessions instead of being integrated throughout the curriculum. The focus is often on whether to teach EBHC or not, rather than on how best to teach and learn EBHC (Hatala 2002, Straus 2000). Consequently, there is a need for better integration and implementation of EBHC teaching and learning throughout the training of doctors, nurses and other healthcare professionals. Many academic institutions are grappling with the challenge of finding the best approach for implementing the teaching and learning of EBHC in student (undergraduate) programmes.

Various systematic reviews have examined the effects of teaching EBHC. This overview will synthesise evidence from systematic reviews of studies of teaching EBHC at undergraduate or post-graduate level and the impact of this teaching on EBHC competencies. It aims to take a systematic approach to gather, evaluate and organise the review-level evidence on teaching EBHC, taking into consideration factors such as type of teaching and target audience, in order to improve access to the evidence and to inform EBHC teaching approaches.

***Research question***

What are the effects of teaching EBHC to health professions at both at both under- and postgraduate levels?

***Objective***

To assess the effects of teaching EBHC to under- and postgraduate health professions’ students.

***Methods***

An update of the overview of systematic reviews will be conducted.

As planned in the initial overview systematic reviews (Cochrane and non-Cochrane) which included randomised trials, quasi-randomised trials, controlled before-and-after studies and interrupted time series will be included. Reviews which evaluated educational interventions (defined as a co-ordinated educational activity, of any medium, duration or format) for teaching EBHC (defined as the process of asking questions, accessing (literature searching), assessing and interpreting research evidence by systematically considering its validity, results and relevance to ones’ own work) to under- and postgraduate health professions students compared to no intervention or a different strategy, will be included. Outcomes will include EBHC knowledge, skills, practices and attitudes, as well as health outcomes. Both under-and postgraduate (specialist training) will be included as strategies used at postgraduate level may inform planning for undergraduate level.

A search in Epistemonikos (Epistemonikos Foundation, Arrayán 2735, Providencia, Santiago, Chile; available at [https://www.epistemonikos.org](https://www.epistemonikos.org/)) will be conducted to identify eligible systematic reviews published from 2013. Adapted MEDLINE search strategy that was used in the original overview will be used for the search in Epistemonikos (Appendix 1). No language restrictions will be used. Ongoing reviews will be identified from searches of PROSPERO, Cochrane Database of Systematic Reviews, JBI Evidence Synthesis, Campbell Library and The Best Evidence Medical Education (BEME) Collaboration. Backwards searching will be conducted to check for potentially eligible reviews not identified through database searching. Experts in the field will be contacted to identify any further reviews. The electronic search results will be exported from Epistemonikos into Covidence (Covidence systematic review software, Veritas Health Innovation, Melbourne, Australia. Available at [www.covidence.org](http://www.covidence.org/)) and screened independently by two researchers for relevance based on the pre-specified eligibility criteria. Full text articles will be obtained of all selected abstracts and will be screened independently for inclusion to determine final study selection. Differences will be resolved by discussion. Researchers working in pairs will independently extract data from each of the included reviews using a predefined and previously piloted data extraction form. Authors of papers will be contacted for missing or additional data. Disagreements will be resolved by discussion.

Included reviews will be summarized in a ‘Characteristics of included systematic reviews’ table.

Two researchers will also independently evaluate the methodological quality of each included systematic review using current version of the 'assessment of multiple systematic reviews' (AMSTAR 2) instrument (Shea 2017). The effect of strategies to teach EBHC will be compared and data will be synthesized and presented in an Overview of reviews table detailing the outcome, intervention and comparison, comparative risks, relative effect and quality of the evidence. If identified systematic reviews for an intervention are more than two years old, a search for more recent studies will be conducted.

***Anticipated benefits***

The research evidence generated from this update of the overview of systematic reviews aims to contribute to enhancing the existing knowledge base regarding the integration of EBHC as a core competency in undergraduate and postgraduate medical education.

***Ethical considerations***

The overview of systematic reviews includes secondary data which are in the public domain and does not involve the enrolment of participants.

# BACKGROUND

## Description of the condition

Evidence-based medicine (EBM), introduced formally as a concept in 1991, has its roots in the field of clinical epidemiology ([Sackett 2002](#REF-Sackett-2002)). The term ‘EBM’ was coined by Gordon Guyatt who defined it as “*an ability to assess the validity and importance of evidence before applying it to day-to-day clinical problems”* ([Dawes 2005](#REF-Dawes-2005)). David Sackett and colleagues (1996) expanded this definition as follows: (EBM is) *“the conscientious, explicit and judicious use of the current best evidence in making decisions about the care of individual patients”.*  EBM thus involves integrating clinical expertise acquired through clinical practice and experience with patient values and current best evidence within the broader healthcare context.

EBM is a systematic approach which includes lifelong self-directed learning in which caring for patients creates the need for important research-based information about clinical and other healthcare issues. Research evidence is constantly changing and therefore, healthcare professionals wishing to provide optimal care need to keep abreast of new developments to be able to offer interventions that work and eliminate the use of those shown to be harmful or ineffective ([Chinnock 2005](#REF-Chinnock-2005)). Practicing EBM promotes critical thinking and typically involves five essential steps: first, converting information needs into answerable questions; second, finding the best evidence with which to answer the questions; third, critically appraising the evidence for its validity and usefulness; fourth, applying the results of the appraisal into clinical practice; and fifth, evaluating performance ([Akobeng 2005](#REF-Akobeng-2005)).

The concept of EBM has also been adopted by many allied healthcare professionals, and the Sicily statement of evidence-based practice ([Dawes 2005](#REF-Dawes-2005)) proposed that the concept of EBM be changed to evidence-based practice (EBP). In the health setting, the term evidence-based health care (EBHC) is often used as it is seen as beneficial for the entire healthcare team, allowing a more holistic, effective approach to health care.

The importance of the knowledge, skills and attitudes acquired through applying the principles of EBHC are also highlighted in the recent Lancet commission report: *Education of health professionals for the 21^st^ century* ([Frenk 2010](#REF-Frenk-2010)), which highlights that the need for healthcare professional training to be transformative. One of the key shifts of transformative learning aligns well with the steps of EBM - the shift from memorization of facts to *“critical reasoning that can guide the capacity to search, analyse, assess and synthesise information for decision-making”* ([Frenk 2010](#REF-Frenk-2010)).

## Description of the interventions

It is widely recommended that EBHC becomes a core component of the curriculum of all healthcare professionals, since learning the fundamentals of research and the how to apply an evidence-based approach fare essential for successful implementation of EBHC and subsequent improvement in the quality of health care ([Glasziou 2008](#REF-Glasziou-2008)).

Various learning and teaching strategies exist. Teaching can be done as standalone sessions or be integrated with clinical practice. It may include journal clubs, bed-side teaching, workshops, lectures, etc. Furthermore it may be offered using face:face contact sessions, online learning or both, and can include both individual and group teaching and learning. The teaching approach may use directed learning or self-directed (problem-based) learning. The content of EBHC curricula is based on the five steps of EBHC. Key competencies required to practice EBHC are detailed in [Figure 1](#FIG-01). A further aspect to factor in is the role of expert teachers and facilitators and their influence on successful learning and teaching in EBHC ([Taheri 2008](#REF-Taheri-2008)).

##

## Figure 1. EBHC competencies

##

## How the interventions might work

Educational activities can impact on EBHC knowledge, skills, attitudes and practice and, ultimately, the quality of health care and outcomes for patients.

Validated tools to assess knowledge and skill acquisition exist and have been widely used ([Shaneyfelt 2006](#REF-Shaneyfelt-2006)), but similar, validated tools to determine the extent to which attitudes change after an educational intervention are lacking. Most studies reporting change in attitude or behaviour rely on student self-reports as measurement tools, but this is not a reliable method for measuring long-term changes in attitude or effects on patient outcomes ([Hatala 2002](#REF-Hatala-2002); [Shaneyfelt 2006](#REF-Shaneyfelt-2006)).

A change in behaviour in the clinical setting and improved patient outcomes are the ultimate outcomes ([Barr 2000](#REF-Barr-2000);[Morrison 2003](#REF-Morrison-2003);[Tavakol 2012](#REF-Tavakol-2012)). Ultimately, this would indicate whether teaching and learning of EBHC was successful. A framework suggested by [Michie 2011](#REF-Michie-2011) describes a *“behaviour change wheel”*, where capability, opportunity and motivation are the three essential conditions that influence behaviour. In applying this to EBHC, capability can be viewed as EBHC knowledge and skills; the opportunity refers to the available resources; while the motivation comes from the individual attitude towards EBHC.

Evaluation of courses should take into account the unique features of medical education. This should include the different settings where learning takes place (bed-side, clinical, remote, outpatient, ambulatory), the background and learning style of the learners, the structure within the courses (for example, large lectures, small groups, one-to-one tuition), and the structure of courses within the larger curriculum (stand-alone courses, integrated teaching) ([Kogan 2007](#REF-Kogan-2007)).

## Why it is important to update the original overview

Various systematic reviews have examined the effects of teaching EBHC, with the evidence on the optimal learning environment, background and learning style of the learners, delivery format, and structure of the most optimal course is still lacking (Kogan 2007). The original overview of systematic reviews assessing the effects of teaching EBHC, published in 2014, concluded that EBHC teaching and learning strategies should be multifaceted, integrated into the clinical context and should include assessment (Young 2014). Since then, a number of systematic reviews addressing questions related to teaching EBHC have been published.

# OBJECTIVES

To update the overview published in 2014 to assess the most recent evidence on the effects of various approaches used in teaching EBHC to healthcare professionals at undergraduate and postgraduate level on changes in knowledge, skills, attitudes and behavior.

# METHODS

## Criteria for considering reviews for inclusion

**Studies**

Systematic reviews (Cochrane and non-Cochrane) which included randomised trials, quasi-randomised trials, controlled before-and-after studies and interrupted time series will be eligible. Eligibility criteria for the original overview were specified as follows: „Systematic reviews were defined as those that had predetermined objectives, predetermined criteria for eligibility, searched at least two data sources, of which one needed to be an electronic database, and performed data extraction and risk of bias assessment.”

For the update systematic reviews will be included if they had predetermined objectives and predetermined eligibility criteria (a protocol), have searched at least two data sources (including at least one electronic database), and have performed data extraction and risk of bias assessment of included studies.

**Participants**

Both undergraduate and postgraduate health professionals (at both student and professional levels) will be eligible.

**Interventions**

Reviews which evaluated any educational intervention (defined as a co-ordinated educational activity, of any medium, duration or format) to teach any component of EBHC (defined as the process of asking questions, accessing (literature searching), assessing and interpreting evidence by systematically considering its validity, results and relevance to ones’ own work) compared to no intervention or a different strategy.

**Outcomes**

EBHC knowledge, skills, attitudes and practice related to EBHC.

##

## Search methods for identification of reviews

Eligible systematic reviews will be searched in the Epistemonikos (Epistemonikos. Epistemonikos Foundation, Arrayán 2735, Providencia, Santiago, Chile; available at [https://www.epistemonikos.org](https://www.epistemonikos.org/)). This database provides a collection of systematic reviews from relevant databases including Cochrane Database of Systematic Reviews, PubMed, EMBASE, CINAHL, PsycInfo, LILACS, Database of Abstracts of Reviews of Effects, The Campbell Collaboration online library, the Johanna-Briggs Institute (JBI) database of Systematic reviews and Implementation Reports, and the EPPI-Centre Evidence Library. The adapted MEDLINE search strategy used in the original overview will be used for the search in Epistemonikos (Appendix 1). No language restrictions will be used. Publication type and Date of publication filters will be applied to restrict the search to systematic reviews published from 1 January 2013. Ongoing reviews will be identified from searches of PROSPERO, Cochrane Database of Systematic Reviews, JBI Evidence Synthesis, Campbell Library and The Best Evidence Medical Education (BEME) Collaboration. Backwards searching will be conducted to check for potentially eligible reviews not identified through database searching.

Search terms will include the following (modified appropriately for the various resources)

1. meta-analysis.mp,pt. OR review.pt OR systematic review.tw.

2. Teaching/ OR teach$.mp OR Education/ OR educa$.mp OR learn$ OR instruct$ OR medical education

3. Evidence Based Practice/ OR evidence based pract$.mp OR Evidence Based Health Care.mp OR Evidence Based Medicine.mp OR EBM.mp

Experts in the field will be contacted and reference lists of included reviews will be checked to identify further reviews that might be eligible.

## Data collection and analysis

### Selection of reviews

Results of the search will be exported from Epistemonikos into Covidence (Covidence systematic review software, Veritas Health Innovation, Melbourne, Australia. Available at [www.covidence.org](http://www.covidence.org/)) and screened for relevance by two authors independently. Full text articles will be obtained of all selected abstracts as well as those where there was disagreement of eligibility to determine final study selection. Differences will be resolved by discussion. The results of the search and selection of studies will be detailed in a flow diagram.

### Data extraction and management

Authors will independently extract data from the eligible reviews by working in pairs and by using a predefined and piloted data extraction form, and included reviews will be summarized in the Characteristics of included reviews table (Table 1). Disagreements will be resolved by discussion.

## Table 1. Characteristics of included reviews table

| Review ID | Aim | Types of participants | Interventions | Comparisons | Studies included | Outcomes | Date recent search |
| --- | --- | --- | --- | --- | --- | --- | --- |
|  |  |  |  |  |  |  |  |
|  |  |  |  |  |  |  |  |

Data to be extracted will include: the key characteristics of reviews, including information about the objectives; participant characteristics; intervention features including content, learning outcomes, teaching strategies, intervention intensities (frequency and duration); setting; outcomes assessed and instruments used to assess outcomes (including its reliability and validity of instruments); comparisons performed and results. We will obtain additional information from the original reports of the studies included in the reviews where necessary.

### Assessment of methodological quality of included reviews

Authors working in pairs will independently evaluate the quality of included reviews. We will resolve differences by discussion and consensus. We will not use the quality of the reviews as inclusion criteria, but we will identify and discuss differences in quality between reviews, and use the review quality assessment to interpret the results of reviews when synthesised in this overview.

### *Quality of included reviews*

The methodological quality of each included review will be assessed using the current version of AMSTAR tool - AMSTAR 2: A MeaSurement Tool to Assess Reviews instrument ([Shea 2017](http://onlinelibrary.wiley.com/o/cochrane/clsysrev/articles/CD007848/bibliography.html#CD007848-bbs2-0078)). AMSTAR assesses the degree to which review methods avoided bias by evaluating the methods reported against 16 distinct criteria. Each item on AMSTAR 2 will be rated as Yes (clearly done), No (clearly not done), and Partial yes. Seven of the 16 items are specified as critical domains: items 2 (protocol), 4 (literature search), 7 (justification for excluding studies), 9 (risk of bias of individual studies), 11 (synthesis methods), 13 (incorporation of risk of bias in interpretation), and 15 (publication bias). The tool has been shown to have good face and content validity for measuring the methodological quality of systematic reviews.These criteria, and the way it will be assessed, are detailed in Table 2. Overall quality of included reviews will be judged by adhering to the published guidance with the following criteria: high – for having none or only one non-critical weakness, moderate - if there was more than one non-critical weakness, low – one critical flaw, critically low – more than one critical flaw (Shea 2017).

Table 2. AMSTAR 2 criteria

| Criteria | |
| --- | --- |
| 1 | Did the research questions and inclusion criteria for the review include the components of PICO? |
| 2 | Did the report of the review contain an explicit statement that the review methods were established prior to conduct of the review and did the report justify any significant deviations from the protocol? |
| 3 | Did the review authors explain their selection of the study designs for inclusion in the review? |
| 4 | Did the review authors use a comprehensive literature search strategy? |
| 5 | Did the review authors perform study selection in duplicate? |
| 6 | Did the review authors perform data extraction in duplicate? |
| 7 | Did the review authors provide a list of excluded studies and justify the exclusions? |
| 8 | Did the review authors describe the included studies in adequate detail? |
| 9a | Did the review authors use a satisfactory technique for assessing the risk of bias (RoB) in individual studies that were included in the review? RCT |
| 9b | Did the review authors use a satisfactory technique for assessing the risk of bias (RoB) in individual studies that were included in the review? nonRCT |
| 10 | Did the review authors report on the sources of funding for the studies included in the review? |
| 11a | . If meta-analysis was performed did the review authors use appropriate methods for statistical combination of results? |
| 11b | If meta-analysis was performed did the review authors use appropriate methods for statistical combination of results? |
| 12 | If meta-analysis was performed, did the review authors assess the potential impact of RoB in individual studies on the results of the meta-analysis or other evidence synthesis? |
| 13 | Did the review authors account for RoB in individual studies when interpreting/ discussing the results of the review? |
| 14 | Did the review authors provide a satisfactory explanation for, and discussion of, any heterogeneity observed in the results of the review? |
| 15 | If they performed quantitative synthesis did the review authors carry out an adequate investigation of publication bias (small study bias) and discuss its likely impact on the results of the review? |
| 16 | Did the review authors report any potential sources of conflict of interest, including any funding they received for conducting the review? |

### Data synthesis

The effect of strategies to teach EBHC will be compared and data will be synthesized and presented in an Overview of review table detailing the outcome, intervention and comparison, comparative risks and relative effect. Descriptive summaries of the included review findings will also be presented.

Odds ratio or risk ratios will be presented for dichotomous outcomes; standardized mean difference (SMD) will be presented for continuous outcomes. Pooled effects will be extracted from the systematic reviews for the available comparisons.

The conceptual framework which will be used will show "what works for whom under which circumstances and to what end" ([Reed 2005](#REF-Reed-2005)).

| What works | Learning objectives, interventions, teaching methods |
| --- | --- |
| Whom | Learners targeted by the intervention |
| Circumstances | Intervention setting, duration, frequency |
| To what end | Desired learner outcomes  Short term – knowledge and awareness  Medium term – attitude  Long term - practice |

#

# CONTRIBUTIONS OF AUTHORS

Taryn Young (TY) developed the protocol. Anke Rohwer (AR) contributed to the background development and provided comments on the methods. Malgorzata M. Bala and Tina Poklepović Peričič adjusted the protocol of the original overview for the purposes of the update.

# DECLARATIONS OF INTEREST

No conflict of interest

## REFERENCES

Akobeng AK. Principles of evidence based medicine. Archives of Disease in Childhood 2005;90:837-40.

Barr H, Freeth D, Hammick M, Koppel, Reeves S. Evaluations of inter professional education: a United Kingdom review of health and social care. London: CAIPE/BERA 2000.

Becker LA, Oxman AD. Chapter 22: Overviews of reviews. In: Higgins JPT, Green S (editors), *Cochrane Handbook for Systematic Reviews of Interventions* Version 5.1.0 (updated March 2011). The Cochrane Collaboration, 2011. Available from www.cochrane-handbook.org.

Chinnock P, Siegfried N, Clarke M. Is Evidence-Based Medicine Relevant to the Developing World? PLoS Med 2005;2(5):e107. doi:10.1371/journal.pmed.0020107.

Dawes M, Summerskill W, Glasziou P, Cartabellotta A, Martin J, Hopayian K, et al. Sicily statement on evidence-based practice. BMC Medical Education 2005;1:5. doi:10.1186/1472-6920-5-1.

Frenk J, Chen L, Bhutta ZA, Cohen J, Crisp N, Evans T, et al. Health professionals for a new century: transforming education to strengthen health systems in an interdependent world. The Lancet 2010;376:1923-58.

Glasziou P, Burls A, Gilbert R. Evidence based medicine and the medical curriculum: The search engine is now as essential as the stethoscope. BMJ 2008;337:704-5.

Hatala R, Guyatt G. Evaluating the Teaching of Evidence-based Medicine. JAMA 2002;288(9):1110-2.

Kogan RJ, Shea AJ. Course evaluation in medical education. Teaching and Teacher Education 2007;23:251-64.

Michie S, van Stralen MM, West R. The behaviour change wheel: A new method for characterising and designing behaviour change interventions. Implementation Science 2011;6(42).

Morrison J. ABC of learning and teaching in medicine: Evaluation. BMJ 2003;326:385-7.

Reed D, Price EG, Windish DM, Wright SM, Gozu A, HsuEB, et al. Challenges in systematic reviews of educational intervention studies. Annals of Internal Medicine 2005;142(12):1080-9.

Sackett DL. Clinical epidemiology: what, who, and whither. Journal of Clinical Epidemiology 2002;55:1161-6.

Shaneyfelt T, Baum KD, Bell D, Feldstein D, Houston TK, Kaatz S, et al. Instruments for evaluating education in evidence-based practice, a systematic review. JAMA 2006;296(9):1116-27.

Shea BJ, **Grimshaw JM,** W**ells GA, Boers M, Andersson N, Hamel C, et al.** Development of AMSTAR: a measurement tool to assess the methodological quality of systematic reviews. BMC Medical Research Methodology 2007;**7**:10 doi:10.1186/1471-2288-7-10

Strauss SE, Green ML, Bell DS, Badgett R, Davis D, Gerrity M, et al. Evaluating the teaching of evidence based medicine: conceptual framework. BMJ 2004;329:1029-32.

Taheri H, Mirmohamadsadeghi M, Adibi I, Ashorion V, Sadeghizade A, Adibi P. Evidence-based Medicine (EBM) for undergraduate medical students. Annals Academy of Medicine 2008;37:764-8.

Tavakol M, Gruppen LD, Torabi S. Using Evaluation research to improve medical education. The Clinical Teacher 2010;7:192-6.

#

# SOURCES OF SUPPORT

## External sources

There will be no external support for conducting this research. The publication fee for this paper will be supported from EU COST Action “EVidence-Based RESearch” number CA-17117.

**Appendix 1**

Teaching EBHC overview: Search adapted for Epistemonikos

1. (title:("evidence-based health care" OR "evidence based health care" OR "evidence-based healthcare" OR "evidence based healthcare" OR EBHC OR EBM OR "evidence-based medicine" OR "evidence based medicine" OR "evidence based nursing" OR "evidence-based nursing" OR EBN OR "evidence-based dentistry" OR "evidence based dentistry" OR EBD OR "evidence-based practice" OR "evidence based practice" OR EBP OR "evidence-based emergency medicine" OR "evidence based emergency medicine" OR "evidence-informed decision-making" OR "evidence informed decision-making" OR "evidence informed decision making" OR EIDM OR "evidence-informed decision making" OR "critical appraisal" OR "journal club") OR abstract:("evidence-based health care" OR "evidence based health care" OR "evidence-based healthcare" OR "evidence based healthcare" OR EBHC OR EBM OR "evidence-based medicine" OR "evidence based medicine" OR "evidence based nursing" OR "evidence-based nursing" OR EBN OR "evidence-based dentistry" OR "evidence based dentistry" OR EBD OR "evidence-based practice" OR "evidence based practice" OR EBP OR "evidence-based emergency medicine" OR "evidence based emergency medicine" OR "evidence-informed decision-making" OR "evidence informed decision-making" OR "evidence informed decision making" OR EIDM OR "evidence-informed decision making" OR "critical appraisal" OR "journal club"))
2. (title:(teach* OR learn* OR course* OR train* OR module* OR workshop* OR curriculum OR curricula OR educate* OR education OR instruct* OR "continuing medical education" OR "continuing professional development" OR "medical education" OR "graduate medical education" OR "undergraduate medical education") OR abstract:(teach* OR learn* OR course* OR train* OR module* OR workshop* OR curriculum OR curricula OR educate* OR education OR instruct* OR "continuing medical education" OR "continuing professional development" OR "medical education" OR "graduate medical education" OR "undergraduate medical education"))
3. #1 AND #2

Filters:

- Publication Type: Systematic review
- Publication year: 2013-2020
