# Supplementary material for: What are the effects of teaching Evidence-Based Health Care (EBHC) at different levels of health professions education? An updated overview of systematic reviews
Source: PLoS One. 2021 Jul 22;16(7):e0254191. doi: 10.1371/journal.pone.0254191 (PMC8297776; doi:10.1371/journal.pone.0254191)
Supplement: S1 Table — (DOCX) [file pone.0254191.s004.docx]

S1.Table. Excluded studies with reasons for exclusion

| **Table. Excluded studies with reasons for exclusion** | |
| --- | --- |
| **Reason** | **Reference to study** |
| Does not meet the definition of systematic review that we used for this overview (no protocol reported)* | 1. Aglen B. Pedagogical strategies to teach bachelor students evidence-based practice: A systematic review. Nurse Educ Today. 2016;1;36:255-63. 2. Albarqouni L, Hoffmann T, Straus S, et al. Core competencies in evidence-based practice for health professionals: consensus statement based on a systematic review and Delphi survey. JAMA Netw Open. 2018;1(2):e180281. 3. Ang RX, Chew QH, Sum MY, et al. Systematic review of the use of debates in health professions education–does it work? GMS J Med Educ. 2019;36(4). 4. Betihavas V, Bridgman H, Kornhaber R, et al. The evidence for ‘flipping out’: A systematic review of the flipped classroom in nursing education. Nurse Educ Today. 2016;38:15-21. 5. Bradd P, Travaglia J, Hayen A. Practice development and allied health -- a review of the literature. International Practice Development Journal. 2017;7(2):1-25. 6. Carter AG, Creedy DK, Sidebotham M. Efficacy of teaching methods used to develop critical thinking in nursing and midwifery undergraduate students: A systematic review of the literature. Nurse Educ Today. 2016;40:209-18. 7. Cui C, Li Y, Geng D, Zhang H, Jin C. The effectiveness of evidence-based nursing on development of nursing students' critical thinking: A meta-analysis. Nurse Educ Today. 2018;65:46-53. 8. Erichsen T, Røkholt G, Utne I. Kunnskapsbasert praksis i sykepleierutdanningen. Norwegian Journal of Clinical Nursing/Sykepleien Forskning. 2016;11(1). 9. Gordon M, Vaz Carneiro A, Patricio M, et al. Missed opportunities in health care education evidence synthesis. Med Educ. 2014;48:644-5. 10. Häggman‐Laitila A, Mattila LR, Melender HL. A systematic review of journal clubs for nurses. Worldviews on Evidence‐Based Nursing. 2016;13(2):163-71. 11. Ilic D, de Voogt A, Oldroyd J. The use of journal clubs to teach evidence‐based medicine to health professionals: A systematic review and meta‐analysis. Journal of Evidence‐Based Medicine. 2020;13(1):42-56. 12. Ilic D, Maloney S. Methods of teaching medical trainees evidence-based medicine: a systematic review. Med Educ. 2014;48(2):124-35. 13. Koota E, Kääriäinen M, Melender H-L. Educational interventions promoting evidence-based practice among emergency nurses: A systematic review. Int Emerg Nurs. 2018;41:51-8. 14. Maggio LA, Tannery NH, Chen HC, et al. Evidence-based medicine training in undergraduate medical education: a review and critique of the literature published 2006-2011. Acad Med. 2013;88(7):1022-8. 15. Melender H-L, Mattila H-L, Häggman-Laitila A. A systematic review on educational interventions for learning and implementing evidence-based practice in nursing education: The state of evidence. Nord J Nurs Res. 2016;36(1):3-12. 16. Spensberger F, Kollar I, Gambrill E, et al. How to Teach Evidence-Based Practice in Social Work: A Systematic Review. Res Soc Work Pract. 2020;30(1):19-39. 17. Upton D, Stephens D, Williams B, et al. Occupational therapists' attitudes, knowledge, and implementation of evidence-based practice: a systematic review of published research. Br J Occup Ther. 2014;77(1):24-38. 18. Wong SC, McEvoy MP, Wiles LK, et al. Magnitude of change in outcomes following entry-level evidence-based practice training: a systematic review. Int J Med Educ. 2013;4:107-14. 19. Ghaffari R, Shapoori S, Binazir MB, et al. Effectiveness of teaching evidence-based nursing to undergraduate nursing students in Iran: a systematic review. Research & Development in Medical Education. 2018;7(1):8-13. 20. Horntvedt MT, Nordsteien A, Fermann T, et al. Strategies for teaching evidence-based practice in nursing education: a thematic literature review. BMC Med Educ. 2018;18(1):172. 21. Safdari R, Ehtesham H, Bahadori L. Highlighting a valuable dimension in health care librarianship: A systematic review. Med J Islam Repub Iran. 2018;32:42. 22. Häggman-Laitila A, Mattila LR, Melender HL. Educational interventions on evidence-based nursing in clinical practice: A systematic review with qualitative analysis. Nurse Educ Today. 2016;43:50-9. 23. Erichsen T, Røkholt G, Utne I. Kunnskapsbasert praksis i sykepleierutdanningen. Norwegian Journal of Clinical Nursing / Sykepleien Forskning. 2018:66-75. 24. Kyriakoulis K, Patelarou A, Laliotis A, et al. Educational strategies for teaching evidence based practice to undergraduate health students: systematic review. J Educ Eval Health Prof. 2016;13:34. 25. Buchanan H, Siegfried N, Jelsma J. Survey Instruments for Knowledge, Skills, Attitudes and Behaviour Related to Evidence-based Practice in Occupational Therapy: A Systematic Review. Occupational therapy international. 2016;23(2):59-90. 26. Galbraith K, Ward A, Heneghan C. A real-world approach to Evidence-Based Medicine in general practice: a competency framework derived from a systematic review and Delphi process. BMC Med Educ. 2017;17(1):78. 27. Im EO, Kong EH. What Is the Status Quo of Evidence-Based Community Health Nursing? Res Theory Nurs Pract. 2017;31(2):156-78. 28. Kamalov M, Dobrynin V, Balykina J, et al. Improving data retrieval quality: Evidence based medicine perspective. Int J Risk Saf Med. 2015;27 Suppl 1:S106-7. 29. Mei-Yeh W, Ching-Chiu K, Chiou-Fen L. The EPCOR Model: A Model for Promoting the Successful Implementation of Evidence-Based Nursing in Hospital-Based Settings. Equipment, Policy, training Courses, Outcome indicators, and Reward plans. J Nurs Res. 2015;23(1):15-24. 30. Roberts MJ, Perera M, Lawrentschuk N, et al. Globalization of Continuing Professional Development by Journal Clubs via Microblogging: A Systematic Review. J Med Internet Res. 2015;17(4):e103. 31. Robson B. Studies in using a universal exchange and inference language for evidence based medicine. Semi-automated learning and reasoning for PICO methodology, systematic review, and environmental epidemiology. Comput Biol Med. 2016;79:299-323. 32. Scurlock-Evans L, Upton D. The Role and Nature of Evidence: A Systematic Review of Social Workers' Evidence-Based Practice Orientation, Attitudes, and Implementation. J Evid Inf Soc Work. 2015;12(4):1-31. 33. Swanberg SM, Dennison CC, Farrell A, et al. Instructional methods used by health sciences librarians to teach evidence-based practice (EBP): a systematic review. J Med Libr Assoc. 2016;104(3):197-208. 34. Yang Y, Liu F, Liu L. Application of evidence-based medicine combined with problem-based learning method in clinical teaching: A meta-analysis. Chinese Journal of Evidence-Based Medicine. 2020: 20(8):962-968. 35. Malicki A, Vergara FH, Van de Castle B, et al. Gamification in Nursing Education: An Integrative Literature Review. J Contin Educ Nurs. 2020;51(11):509-515. 36. Patelarou AE, Kyriakoulis KG, Stamou AA, et al. Approaches to teach evidence-based practice among health professionals: an overview of the existing evidence. Adv Med Educ Pract. 2017;8:455-64. |
| Not intervention of interest | 1. Barrett J, Gonsalvez CJ, Shires A. Evidence‐based practice within supervision during psychology practitioner training: A systematic review. Clin Psychol (Aust Psychol Soc). 2019. 2. Kumaravel, B., Hearn, J.H., Jahangiri, L. et al. A systematic review and taxonomy of tools for evaluating evidence-based medicine teaching in medical education. Syst Rev 9, 91 (2020):https://doi.org/10.1186/s13643-020-01311-y 3. Li S, Cao M, Zhu X. Evidence-based practice: Knowledge, attitudes, implementation, facilitators, and barriers among community nurses-systematic review. Medicine. 2019;98(39):e17209. 4. Ramis MA, Chang A, Nissen L. Undergraduate Health Students' Intention to Use Evidence-Based Practice After Graduation: A Systematic Review of Predictive Modeling Studies. Worldviews Evid Based Nurs. 2018;15(2):140-8. 5. Simons MR, Zurynski Y, Cullis J, Morgan MK, Davidson AS. Does evidence-based medicine training improve doctors' knowledge, practice and patient outcomes? A systematic review of the evidence. Med Teach. 2019;41(5):1-7. 6. Thomas RE, Kreptul D. Systematic review of evidence-based medicine tests for family physician residents. Fam Med. 2015;47(2):101-17. 7. Pedrosa KKA, Oliveira ICM, Feijão AR,et al. Evidence-based nursing: Characteristics of studies in brazil. Cogitare enfermagem. 2015;20(4):01-8. 8. Pedersen ER, Kandrack R, Danz M, et al. Provider Interventions to Increase Uptake of Evidence-Based Treatment for Depression: A Systematic Review. Rand Health Q. 2020;9(1):6. 9. Connolly P, Keenan C, Urbanska K. The trials of evidence-based practice in education: A systematic review of randomised controlled trials in education research: Correction. Educational Research. 2018;60(4) (Correction of the article: Connolly P, Keenan C, Urbanska K. The trials of evidence-based practice in education: a systematic review of randomised controlled trials in education research 1980–2016, Educational Research. 2018;60:3, 276-291) 10. Silva AMd, Comper ML, Costa LdCM, et al. Instrument for assessing evidence-based practice in physical therapy: a systematic review. Conscientiae saúde (Impr). 2015;14(2):321-7. |
| Not outcome of interest | 1. Albarqouni L, Hoffmann T, Glasziou P. Evidence-based practice educational intervention studies: a systematic review of what is taught and how it is measured. BMC Med Educ. 2018;18(1):177. 2. Valenstein-Mah H, Greer N, McKenzie L, et al. Effectiveness of training methods for delivery of evidence-based psychotherapies: a systematic review. Implement Sci. 2020;15(1):40. |
| Not population of interest | 1. Sarkies MN, Bowles KA, Skinner EH, et al. The effectiveness of research implementation strategies for promoting evidence-informed policy and management decisions in healthcare: a systematic review. Implement Sci. 2017;12(1):132. 2. Mikkonen K, Ojala T, Sjögren T, et al. Competence areas of health science teachers - A systematic review of quantitative studies. Nurse Educ Today. 2018;70:77-86. |
| Excluded from original overview |  |
| Does not meet definition of systematic review* | 1. Alguire PC. A review of journal clubs in postgraduate medical education. J Gen Intern Med. 1998;13: 347-353. 2. Mi M. Evidence Based Medicine Teaching in Undergraduate Medical Education: A Literature Review. Evid Based Libr Inf Pract. 2012; 7. 3. Werb SB, Matear DW. Implementing evidence-based practice in undergraduate teaching clinics: a systematic review and recommendations. J Dent Educ. 2004;68: 995-1003. |
| Not outcome of interest | 1. Malick SM, Hadley J, Davis J, et al. Is evidence-based medicine teaching and learning directed at improving practice? J R Soc Med. 2010;103: 231-238. |
| *i.e. predetermined objectives and predetermined criteria for eligibility (for an update specified as having protocol), have searched at least two data sources (including at least one electronic database), and have performed data extraction and risk of bias assessment) | |
