# Supplementary material for: What are the effects of teaching Evidence-Based Health Care (EBHC) at different levels of health professions education? An updated overview of systematic reviews
Source: PLoS One. 2021 Jul 22;16(7):e0254191. doi: 10.1371/journal.pone.0254191 (PMC8297776; doi:10.1371/journal.pone.0254191)
Supplement: S2 Checklist — (DOCX) [file pone.0254191.s005.docx]

| the Synthesis Without Meta-analysis (SWIM) checklist reporting for EBHC overview  Campbell M, McKenzie JE, Sowden A, Katikireddi SV, Brennan SE, Ellis S, Hartmann-Boyce J, Ryan R, Shepperd S, Thomas J, Welch V, Thomson H. Synthesis without meta-analysis (SWiM) in systematic reviews: reporting guideline BMJ 2020;368:l6890 http://dx.doi.org/10.1136/bmj.l6890 | | | |
| --- | --- | --- | --- |
| **SWiM is intended to complement and be used as an extension to PRISMA** | | | |
| **SWiM reporting**  **item** | **Item description** | **Page in manuscript**  **where item is reported** | Other |
| Methods |  |  |  |
| **1** Grouping  studies for  synthesis | 1a) Provide a description of, and rationale for, the groups used in the synthesis (e.g., groupings of  populations, interventions, outcomes, study design) | Page 10-12 |  |
|  | 1b) Detail and provide rationale for any changes made subsequent to the protocol in the groups used  in the synthesis | NA |  |
| **2** Describe the  standardised  metric and  transformation  methods used | Describe the standardised metric for each outcome. Explain why the metric(s) was chosen, and  describe any methods used to transform the intervention effects, as reported in the study, to the  standardised metric, citing any methodological guidance consulted | NA | The majority of review did not provide numerical data |
| **3** Describe the  synthesis  methods | Describe and justify the methods used to synthesise the effects for each outcome when it was not  possible to undertake a meta-analysis of effect estimates | Page 10-11 |  |
| **4** Criteria used  to prioritise  results for  summary and  synthesis | Where applicable, provide the criteria used, with supporting justification, to select the particular  studies, or a particular study, for the main synthesis or to draw conclusions from the synthesis (e.g.,  based on study design, risk of bias assessments, directness in relation to the review question) | Page 10-11 | All included reported on |
| **5** Investigation  of  heterogeneity in  reported effects | State the method(s) used to examine heterogeneity in reported effects when it was not possible to  undertake a meta-analysis of effect estimates and its extensions to investigate heterogeneity | Page 10-11 |  |
| **6** Certainty of  evidence | Describe the methods used to assess certainty of the synthesis findings | NA | According to the guidance form Cochrane Handbook reported as in the SRs included |
| **7** Data  presentation  methods | Describe the graphical and tabular methods used to present the effects (e.g., tables, forest plots,  harvest plots).  Specify key study characteristics (e.g., study design, risk of bias) used to order the studies, in the text  and any tables or graphs, clearly referencing the studies included | Page 10-11 |  |
| Results |  |  |  |
| **8** Reporting  results | For each comparison and outcome, provide a description of the synthesised findings, and the  certainty of the findings. Describe the result in language that is consistent with the question the  synthesis addresses, and indicate which studies contribute to the synthesis | Page 27-31, Table 3 A and 3B; File S6, S7, S8; Page 37-39, fig 4-6 | According to the guidance form Cochrane Handbook reported as in the SRs included (only 2 reported) |
| Discussion |  |  |  |
| **9** Limitations of  the synthesis | Report the limitations of the synthesis methods used and/or the groupings used in the synthesis, and  how these affect the conclusions that can be drawn in relation to the original review question | Page 41-43 |  |
