# Supplementary material for: What are the effects of teaching Evidence-Based Health Care (EBHC) at different levels of health professions education? An updated overview of systematic reviews
Source: PLoS One. 2021 Jul 22;16(7):e0254191. doi: 10.1371/journal.pone.0254191 (PMC8297776; doi:10.1371/journal.pone.0254191)
Supplement: S2 File — (DOCX) [file pone.0254191.s007.docx]

S2 File. MEDLINE and Epistemonikos search strategies

**1. Search strategy used in the update of the overview in Epistemonikos database**

(MEDLINE Search strategy adapted for Epistemonikos) (October 2020)

1. (title:("evidence-based health care" OR "evidence based health care" OR "evidence-based healthcare" OR "evidence based healthcare" OR EBHC OR EBM OR "evidence-based medicine" OR "evidence based medicine" OR "evidence based nursing" OR "evidence-based nursing" OR EBN OR "evidence-based dentistry" OR "evidence based dentistry" OR EBD OR "evidence-based practice" OR "evidence based practice" OR EBP OR "evidence-based emergency medicine" OR "evidence based emergency medicine" OR "evidence-informed decision-making" OR "evidence informed decision-making" OR "evidence informed decision making" OR EIDM OR "evidence-informed decision making" OR "critical appraisal" OR "journal club") OR abstract:("evidence-based health care" OR "evidence based health care" OR "evidence-based healthcare" OR "evidence based healthcare" OR EBHC OR EBM OR "evidence-based medicine" OR "evidence based medicine" OR "evidence based nursing" OR "evidence-based nursing" OR EBN OR "evidence-based dentistry" OR "evidence based dentistry" OR EBD OR "evidence-based practice" OR "evidence based practice" OR EBP OR "evidence-based emergency medicine" OR "evidence based emergency medicine" OR "evidence-informed decision-making" OR "evidence informed decision-making" OR "evidence informed decision making" OR EIDM OR "evidence-informed decision making" OR "critical appraisal" OR "journal club"))
2. (title:(teach* OR learn* OR course* OR train* OR module* OR workshop* OR curriculum OR curricula OR educate* OR education OR instruct* OR "continuing medical education" OR "continuing professional development" OR "medical education" OR "graduate medical education" OR "undergraduate medical education") OR abstract:(teach* OR learn* OR course* OR train* OR module* OR workshop* OR curriculum OR curricula OR educate* OR education OR instruct* OR "continuing medical education" OR "continuing professional development" OR "medical education" OR "graduate medical education" OR "undergraduate medical education"))
3. #1 AND #2

Filters:

- Publication Type: Systematic review
- Publication year: 2013-2020

Total hits: 894

**2. The search strategy in MEDLINE (via Ovid) for original 2014 overview**

Database: Ovid MEDLINE(R) and Epub Ahead of Print, In-Process & Other Non-Indexed Citations and Daily <1946 to September 13, 2019>

Search Strategy:

--------------------------------------------------------------------------------

1 exp Evidence-Based Practice/ (85289)

2 evidence-based medicine.tw. (12885)

3 evidence-based practice.tw. (9158)

4 evidence-based research.tw. (788)

5 evidence-based health care.tw. (429)

6 evidence-based healthcare.tw. (269)

7 evidence informed health care.tw. (8)

8 evidence informed healthcare.tw. (15)

9 evidence-based decision making.tw. (986)

10 (EBM or EBP or EBR or EBHC).tw. (11399)

11 or/1-10 (104370)

12 exp Education, Professional/ (288999)

13 exp Teaching/ (82610)

14 exp Curriculum/ (81608)

15 exp Learning/ (369847)

16 (educat$ or teach$ or train$ or learn$ or motivat$ or instruct$ or interven$ or promot$).tw. (3048739)

17 (course$ or seminar$ or workshop$).tw. (628902)

18 (pedagogic$ adj3 (approach$ or method$ or modalit$)).tw. (846)

19 or/12-18 (3880579)

20 11 and 19 (36053)

21 limit 20 to (meta analysis or "systematic review") (2627)

***************************
