# Supplementary material for: What are the effects of teaching Evidence-Based Health Care (EBHC) at different levels of health professions education? An updated overview of systematic reviews
Source: PLoS One. 2021 Jul 22;16(7):e0254191. doi: 10.1371/journal.pone.0254191 (PMC8297776; doi:10.1371/journal.pone.0254191)
Supplement: S3 File — (DOCX) [file pone.0254191.s008.docx]

### S3 File. Description of the results of newly identified reviews.

Detailed information about the 6 newly identified reviews[1-6]

### Methodological quality of systematic reviews

Among the newly identified SRs, one was judged as having high quality, [5] one as having low quality [1] and four as having critically low quality [2-4, 6]. The most common reasons for low quality were lack of a comprehensive search, not providing a list of excluded studies with justification for exclusion, and not using satisfactory risk of bias assessment in individual studies included in the review.

### Effects of interventions

Ramis et al (critically low quality) assessed the effects of teaching EBHC to undergraduate students on EBHC knowledge, skills, attitudes, use and future use, and reported a small to moderate significant increase in EBHC knowledge scores compared to a control group (two studies) or to pre-test score (one study) [4]. Three out of four included studies reported significant improvements in EBHC attitude, one compared to a control group in the short term, and two compared to pre-test scores in the longer-term (two studies). One study found no difference in EBHC attitude compared to a control group in the short term. EBHC skills improved compared to a control group in the short term (one study) and compared to pre-test scores (2 studies) in the long term. One study reported a small significant difference for EBHC use in the intervention compared to the control group, but no significant difference for future EBHC use.

Four of the new SRs [1-3, 6] included postgraduates only (all critically low quality) and assessed the effectiveness of EBHC skills training for various types of healthcare professionals. Hecht et al also assessed quality of reporting of the development and implementation of complex interventions. The studies included in the Hecht review showed small improvements in knowledge and skills and self-perceived implementation of EBHC [2]. Hines et al (critically low quality) compared a variety of educational interventions delivered to post-registration nurses in a workplace environment, including journal clubs, short workshops, long-term courses (such as a six-week research knowledge course or six-month clinical fellowship program); or different course formats in a university environment (such as virtual, self-directed, face-to-face, etc.) delivered to post-registration nurses [3]. They showed improvements in EBHC knowledge and critical appraisal skills in face-to-face and online intervention groups compared to control groups and pre-test scores; and no difference between the groups receiving the same course using traditional lectures or the interactive online modules. Two reviews evaluated the effect of EBHC training on patient outcomes or process of care [1, 6]. The review by Wu (critically low quality) assessed the use of a variety of single and multifaceted educational interventions in postgraduate and continued medical education training based on EBHC principles [6]. They narratively reported positive project-related patient outcomes in 15 of the 18 included studies and mixed project-related patient outcomes in one study. Due to heterogeneity in interventions and outcomes they did not combine the results in meta-analyses. In 12 studies, which provided details with the examples, there were improvements in symptom management (pain) and rates of complications (pressure ulcers, catheter or central-line related infections, aspiration pneumonia, ventilator-associated pneumonia, patients’ anxiety, length of hospitalization, costs). In four studies, some benefits were discussed but according to the review authors incomplete data were provided in primary studies. Fiander et al (low quality) assessed interventions that encouraged practitioners (physicians, residents, allied health professionals) to use electronic health information (EHI) to improve clinical practice and patient outcomes [1]. The review reported the overall certainty of evidence to be rated as low. Fiander et al did not conduct meta-analysis due to heterogeneity in comparisons, outcomes, and reporting. Three out of six eligible studies included multifaceted group education in the use of electronic health information and reported a positive effect of multifaceted interventions to specifically increase the use of EHI, but it was unclear which aspect of the interventions influenced practitioners’ behaviour. However, the SR found no evidence that the use of EHI translates into improved clinical practice or patient outcomes [1].

Rohwer et al (high quality) assessed pure EBHC e-learning, as well as blended learning compared to no EBHC learning, only face-to-face learning, or pure e-learning including different components on EBHC knowledge, attitude and behaviour in a mixed population of under- and postgraduates [5]. For the comparison of pure e-learning versus no EBHC learning, the results favoured e-learning with moderately higher knowledge scores and a large increase in EBHC attitude. There was no difference for knowledge and skills as a composite outcome and there was some evidence on improvement in skills. For the comparison of blended learning versus no EBHC learning, the results favoured blended learning with moderately higher knowledge scores, large increases in knowledge and skills as a composite outcome one and three months after the intervention, mixed results regarding EBHC attitude, with one study reporting an effect directly after the intervention but not in the longer term (three months). The results for EBHC behaviour were mixed by time: no difference was observed between blended learning and no learning in the studies which measured the effect immediately after and one month post-intervention, while studies assessing the effect at least three months after the intervention showed a significant medium sized effect favouring blended learning. For the comparison of pure e-learning compared to face-to-face EBHC learning, there was no difference in EBHC knowledge, skills and attitude. For the comparison of blended versus face-to-face learning, the review found similar effects for EBHC knowledge and skills, but results favoured blended learning for attitude and behaviour. For the comparison of blended learning versus pure e-learning, the results favoured blended learning showing higher knowledge scores, but mixed results for EBHC skills, whereas one randomized trial favoured blended learning and one non-randomized trial favoured pure e-learning. For the comparison of two pure e-learning strategies with different components in two studies, the review reported higher skills scores for the groups, who received a DVD as compared to standard distance learning and online journal club as compared to access to journal articles via e-mail only. One study that compared three different strategies for AGREEII tutorials did not show any differences between the groups.

1. Fiander, M., et al., *Interventions to increase the use of electronic health information by healthcare practitioners to improve clinical practice and patient outcomes.* Cochrane Database Syst Rev, 2015. **3**(3).

2. Hecht, L., S. Buhse, and G. Meyer, *Effectiveness of training in evidence-based medicine skills for healthcare professionals: a systematic review.* BMC Med Educ, 2016. **16**(1): p. 103.

3. Hines, S., J. Ramsbotham, and F. Coyer, *Interventions for improving the research literacy of nurses: a systematic review.* JBI Database System Rev Implement Rep, 2016. **14**(2): p. 256-94.

4. Ramis, M.A., et al., *Theory-based strategies for teaching evidence-based practice to undergraduate health students: a systematic review.* BMC Med Educ, 2019. **19**(1): p. 267.

5. Rohwer, A., et al., *E-Learning of Evidence-Based Health Care (EBHC) in Healthcare Professionals: A Systematic Review. Campbell Systematic Reviews 2017: 4.* Campbell Collaboration, 2017.

6. Wu, Y., et al., *Do educational interventions aimed at nurses to support the implementation of evidence-based practice improve patient outcomes? A systematic review.* Nurse Educ Today, 2018. **70**: p. 109-114.
